# Role of oxygen inhibited layer on shear bond strength of composites

**DOI:** 10.4103/0972-0707.62635

**Published:** 2010

**Authors:** Sheetal Ghivari, Manoj Chandak, Narendra Manvar

**Affiliations:** Department of Conservative Dentistry and Endodontics, Sharad Pawar Dental College and Hospital Sawangi (M), Wardha, India

**Keywords:** Composites, shear bond strength, oxygen inhibited layer

## Abstract

**Background and Aim:**

Rising demand for aesthetic adhesive restorations has led to wide use of composites. A multilayer technique is recommended for success of these restorations. The surface layer of composite coming in contact with air forms a superficial sticky layer called oxygen inhibited layer, upon polymerization, allowing resins from both sides to cross the interface and form an interdiffusion zone. The present study was sought to test whether oxygen inhibited layer increases or decreases the shear bond strength at the interface of composites.

**Materials and Methods:**

A microhybrid composite Esthetic –X (Dentsply, Caulk) was used in this study. A cylindrical mold of composite, five mm thick and eight mm long, was prepared and embedded in acrylic resin molds after curing. This was placed in distilled water for two hours and sheared in universal testing machine at a cross head speed of one mm/sec.

**Statistical Analysis:**

Data analyzed statistically to determine the significant difference between the groups. Mean and standard deviation values were estimated for the study groups and compared by one way ANOVA.

**Results:**

No significant difference in shear bond strength of composites cured with and with out oxygen inhibited layer.

**Conclusions:**

The presence or absence of oxygen inhibited layer made no significant difference in shear bond strength of composite resins.

## INTRODUCTION

Typically, dental composites are random copolymers of Bis – GMA [4,-(2, hydroxy-3-methacryloxypropoxy) phenyl] propane (Bis-GMA), triethyleneglycol dimethacrylate (TEDGMA) and filled with various inorganic filler particles. Bis-GMA and TEGMA are bifunctional monomers that harden by free radicle induced polymerization. This reaction is strongly inhibited by free radicle scavengers such as oxygen.[1,2] The inhibition resulting from oxygen diffusing from atmosphere into curing resin is responsible for formation of a soft, sticky superficial layer on freshly polymerized resin, referred to as oxygen inhibited layer.[2–4]

Oxygen inhibited layer is always present when bonding agent or composite is polymerized in air. The oxygen inhibited layer is primarily composed of unreacted monomers and oligomers and possesses a liquid-like consistency. This layer not only readily adopts the overlying material to increase contacting area but also allows materials on both sides to cross the interface and blend together to form an interdiffused zone where copolymerization can take place to produce a chemical bond. All these actions will tend to strengthen layer-layer interaction.[5]

For years it was a common perception that an oxygen- inhibited layer is required before adding more layers of bonded composite. Based on the principle of molecular interaction, one might easily reason that an oxygen-inhibited layer should improve the interfacial bonding between two contacting polymers.[6–7]

Reports on how the oxygen-inhibited layer affects bond strength have been inconsistent. A few studies reported that the presence of an oxygen-inhibited layer made no significant differences to bond strength.[8–9]

This study aimed to evaluate whether the presence of oxygen inhibited layer increases or decreases the shear bond strength.

## MATERIALS AND METHODS

Resin composites used in this study were Esthetic–X (Dentsply, Caulk) which is micro hybrid and samples were cured in Q-Lux (Dentsply) curing unit for 20 seconds per layer. Sixteen composite samples with standard dimensions of five mm (diameter), and eight mm (height) were prepared.

Samples were embedded in acrylic resin blocks so that the interface of two increments is at resin substrate junction to facilitate testing in universal testing machine. Each sample consisted of two layers of four mm each cured for 20 seconds.

Samples were divided into two groups of eight samples each.

Group 1 (n is equal to eight) – Presence of oxygen inhibited layer.

Samples were prepared by placing first increment of composite in a gelatine capsule without cellophane strip and cured, subsequent layer of composite added by pushing the first increment in gelatine capsule so that oxygen inhibited layer is in between the subsequent increments of four mm.

Group 2 (n = 8) – Absence of oxygen inhibited layer.

Samples were prepared as above and cured in gelatine capsule with cellophane strip between the subsequent increments of 4 mm.

After photo polymerization the samples were conditioned in distilled water for two hours and specimens were sheared to failure in universal testing machine (Model 4466, Instron Inc., Canton, MA, USA) at cross head speed of 1 mm/min.

Bond strength calculated = F (force)/A (area).

A = Πr^2^,; A= cross sectional area of interface, r = Radius of sample.

The average bond strength for each group was calculated.

## RESULTS

**Table:** 

Group	n	mean	Std. deviation	Std. error mean	*T* value	*P* value
1	8	6.21	0.65	0.23	1.46	0.16
2	8	5.61	0.97	0.97		NS, *P*>0.05

Data was analyzed between both the groups to determine the significant difference between them. Mean and standard deviation values were estimated for the study groups and compared by one way ANOVA.

Group 1 consisting of composite samples cured with oxygen inhibited layer showed no significant difference in shear bond strength compared to group 2 cured without oxygen inhibited layer (NS, *P* > 0.05) at the interface between the increments.

## DISCUSSION

Dental composites are widely used esthetic restorative materials in dentistry. To minimize polymerization shrinkage and increase the degree of conversion, multilayer technique is recommended for ultimate success of composite; bond strength between different layers becomes important.^[13,14]^ However, during polymerization the free oxygen in contact with composite resin diffuses and inhibits polymerization reaction forming peroxide radicals which have low reactivity towards monomers. The free monomer layer will remain on the surface after curing as reactivity of oxygen is much higher with radicle than with monomer. This free monomer layer remaining on the surface after curing is known as oxygen inhibited layer and always formed when composites are polymerized in presence of air.

$\text{R}+{\text{O}}_{2}\to \text{R}-\text{OO}\left(\text{stable radicals}\right)$

Studies have shown a positive correlation indicating oxygen inhibited layer increases bond strength by formation of covalent bond within interpenetrating network.[6,7] However, a few studies state that oxygen inhibited layer is detrimental to bond strength due to its brittleness.[10–12] Some recent studies have concluded that oxygen inhibited layer made no significant difference in bond strength.[8,3]

In the present study shear bond strength of composite resin (Esthetic–X) was measured with or without oxygen inhibited layer. It is found that presence of oxygen inhibited layer made no significant difference in shear bond strength. The resin in the oxygen inhibited layer has the same composition as the uncured resin, except that the photoinitiator system, commonly camphorquinone (CQ) and amine, has been consumed or decomposed.[10] The rate of CQ decomposition was found to be irradiation energy dependant. A comparison of results showed that the CQ half-life decreases with higher-intensity irradiation. The oxygen inhibited layer contains significantly reduced levels of CQ photo initiator. They also imply that a long curing time may negatively impact the ability of the oxygen inhibited layer to be post-cured. Extremely high thickness of the oxygen-inhibited layer of traditional composites (37 plus/minus16 mm) prevented effective interdiffusion of fresh composite (photoinitiator) into the inhibited layer, resulting in under polymerization and lower bond strength.[5]

### Esthetics-X

X hybrid composite used in present study composed of Phenyl propandion (PPD) as initiator and filler particle size is smaller compared to traditional macrofilled composites. It has been found that interfacial bond strength decreases as filler loading changes from highly filled to microfilled.[12] Increased oxygen solubility of uncured resin due to absorption of oxygen on to surface of filler particles may provoke decrease in conversion at composite/atmosphere interface this confirm filler particles may influence bond strength. According to a study by Byoung, with advancement in composite material and photoinitiator systems (nanofilled composites) the thickness of the oxygen-inhibited layer was recently measured to be much less than six μm (Bisco data on file). With a lower thickness of oxygen inhibited layer, it is possible to completely interdiffuse the oxygen-inhibited layer with fresh composite overlay, resulting in normal bond strength.[5]

This study used micro hybrid composite with improved photoinitiator and reduced filler particle size as compared to conventional composites, reducing the thickness of oxygen inhibited layer by complete interdiffusion of oxygen inhibited layer with fresh composite overlay.

Taking these observations into consideration it can be concluded that the presence or absence of oxygen inhibited layer does not have influence on bond strength.

## CONCLUSION

Under the limitations of the present study and contrary to common perception, it can be concluded that the presence of oxygen inhibited layer made no significant difference in shear bond strength of composite resins.
